# Kinematic analysis of movement impaired by generalization of fear of movement-related pain in workers with low back pain

**DOI:** 10.1371/journal.pone.0257231

**Published:** 2021-09-17

**Authors:** Ren Fujii, Ryota Imai, Shinichiro Tanaka, Shu Morioka

**Affiliations:** 1 Department of Neurorehabilitation, Graduate School of Health Science, Kio University, Kitakatsuragi-gun, Japan; 2 Department of Rehabilitation, Medical Corporation Tanakakai, Musashigaoka Hospital, Kumamoto-shi, Japan; 3 School of Rehabilitation, Osaka Kawasaki Rehabilitation University, Kaizuka-shi, Japan; 4 Department of Rehabilitation Medicine, Medical Corporation Tanakakai, Musashigaoka Hospital, Kumamoto-shi, Japan; 5 Neurorehabilitation Research Center, Kio University, Kitakatsuragi-gun, Japan; Toronto Rehabilitation Institute - UHN, CANADA

## Abstract

**Purpose:**

To identify impaired trunk movement during work-related activity in individuals with low back pain (LBP) and investigate whether abnormalities were caused by generalized fear of movement-related pain.

**Methods:**

This cross-sectional study was conducted at a hospital in Japan. We recruited 35 participants with LBP (LBP group; 26 males, 9 females) and 20 healthy controls (HC group) via posters at our hospital. The task required lifting an object. We used a 3D motion capture system to calculate the peak angular velocity of trunk flexion and extension during a lifting task. Pain-related factors for the LBP group were assessed using the visual analogue scale (VAS) for pain intensity over the past 4 weeks and during the task, the Tampa Scale for Kinesiophobia (TSK), the Pain Catastrophizing Scale (PCS), and the Pain Anxiety Symptoms Scale-20 (PASS-20). We compared kinematic variables between groups with a generalized linear mixed model and investigated the relationship between kinematic variables, VAS scores, and psychological factors by performing a mediation analysis.

**Results:**

The peak angular velocity of trunk extension showed significant main effects on the group factors (LBP group vs. HC group) and their interactions; the value of the kinematic variable was lower at Trial 1 in the LBP group. No LBP participant reported pain during the experiment. The mediation analysis revealed that the relationship between the VAS score for pain intensity over the past 4 weeks and the peak angular velocity of trunk extension in the first trial was completely mediated by the TSK (complete mediation model, 95% bootstrapped CI: 0.07–0.56).

**Conclusion:**

Individuals with LBP had reduced trunk extension during a lifting task. Generalized fear of movement-related pain may contribute to such impaired trunk movement. Our findings suggest that intervention to ameliorate fear of movement may be needed to improve LBP-associated disability.

## Introduction

Occupational low back pain (LBP) is a serious health problem in many industrialized countries [1]; it has a negative impact on work disability and labor productivity and causes great losses to the social economy [2]. LBP is the most frequent occupational health problem, with 80%–90% of workers worldwide experiencing some degree of occupational LBP [3]. Epidemiological studies from various countries have shown a lifetime LBP prevalence of approx. 80% [4, 5] and revealed that ~50% of workers who experience LBP continue to work with LBP [6].

Occupational LBP often causes impaired trunk movement, including limited trunk movement range and velocity [7], abnormal trunk muscle contraction [8], and decreased lumbo-pelvic coordination [9]. The risk factors for occupational LBP include both ’motor’ factors (e.g., high physical demands of work, physical dysfunction, and impaired movement) [5], and ’psychosocial’ factors (e.g., fear, anxiety, and catastrophic thinking) [10, 11]. A relationship has been observed between occupational LBP and the physical demands of work, and the rehabilitation for LBP has thus focused on the ’motor’ aspect. However, ergonomic interventions alone have been shown to be insufficient [12].

In other words, because occupational LBP is influenced by psychosocial factors, the motor and psychosocial dysfunctions are thought to overlap [10]. Among the possible psychological factors in LBP, a systematic review of musculoskeletal diseases reported that fear of movement is a risk factor that strongly affects the symptoms of chronic pain [13]. Investigations of patients with chronic LBP reported that fear of movement affects the prognoses of dysfunction and disability [14] and influences both chronic and occupational LBP [15]. However, the mechanisms underlying the impairment of trunk movement in occupational LBP remain unclear.

Meulders proposed that one of the causes of fear of movement is the generalization of fear of movement-related pain [16]. Such generalized fear is evoked by both specific movements that cause pain and similar perceived physical threats related to contextually relevant movement. For example, when a person bends to pick something up and pain is experienced, fear of that movement is evoked; fear of similar movements that might also cause pain (e.g., trunk bending with a smaller movement range) is also evoked. Thus, generalized fear of movement-related pain causes impaired movement, even when the contemplated movement does not cause pain.

Many workers with occupational LBP are able to continue working, and their occupational LBP is thus considered to be less severe, and their pain intensity mild [17]. Generalization of fear of movement-related pain may therefore be the cause of the impaired trunk movement rather than actual pain that occurs during work-related activity. It is therefore necessary to analyze in detail whether fear of movement is triggered by actual pain caused during movement and/or by a past pain experience of pain caused during movement. Studies of relationships between psychological factors and kinematic characteristics revealed that fear of movement is associated with slower movement, but no causal relationship has been established among pain characteristics (e.g., pain during movement vs. a memory of past pain), fear of movement, and kinematic features [18, 19]. Revealing how pain-related factors cause impaired trunk movement could be important for the comprehensive management of workers with occupational LBP.

Although the mechanisms of impaired trunk movement in occupational LBP are unclear, impaired trunk movement during work-related activity in people with LBP was reported to be characterized by a limitation of the movement velocity of the trunk to minimize pain caused by mechanical stress [20]. We hypothesized that such impairment of movement is influenced not by sensory pain during movement; rather, the impairment is influenced by generalized fear of movement-related pain. We conducted the present study to investigate whether impaired trunk movement is caused by generalization of fear of movement-related pain based on past pain experiences—i.e., whether fear of movement mediates the relationship between pain intensity and impaired trunk movement.

## Methods

### Study design and participants

The participants were nurses, caregivers, and rehabilitation specialists at our hospital, recruited via posters stating the inclusion and exclusion criteria. The inclusion criteria for the LBP group were chosen with reference to [21] and were as follows: (1) pain that occurred from the lower rib edge to the gluteal fold for >1 day; (2) LBP that occurred when lifting a heavy object during work; (3) LBP duration of >3 months; and (4) a score of ≥10 mm on a visual analogue scale (VAS) for pain intensity. Fifty participants with LBP met all of these criteria; 15 of them met one of the following exclusion criteria and were excluded: (1) a previous diagnosis of spinal disease (n = 9); (2) the presence of neurological symptoms of a lower limb (n = 3); (3) pain in a limb joints other than the lumbar region (n = 3); and (4) a previous diagnosis of a neurological disorder, such as a cerebrovascular disorder (n = 0). Thus, 35 participants comprised the study LBP group. We also used posters to recruit 20 age-matched healthy controls with no history of LBP and no other diagnosed illnesses (HC group).

The study protocol conformed to the Declaration of Helsinki. We explained the study in advance to the participants orally and in writing and obtained their written consent. The participants were allowed to withdraw their consent and participation at any time, even after consenting (e.g., if a participant’s LBP worsened before or during the experimental task, the experiment was stopped immediately). The study was also approved by the ethics committee of Kio University Health Sciences Graduate School (approval no. H30-04).

### Procedure

This study used a cross-sectional design. Participants were asked to respond to the VAS about the maximum pain they had experienced during the past 4 weeks, and to complete the Tampa Scale for Kinesiophobia (TSK) [22], the Pain Catastrophizing Scale (PCS) [23], the Pain Anxiety Symptoms Scale-20 (PASS-20) [24], the Roland-Morris Disability Questionnaire (RDQ) [25], and Von Korff’s grading for the severity of LBP [26] for the assessment of pain-related indicators before they completed the experimental task.

After the pain-related indicator assessment, each participant performed the experimental task, which involved lifting an object five times at 1-min intervals. Further details are provided below. We adopted the lifting task because it is a work-related activity that is widely recognized as a risk factor for occupational LBP [27] and has been used as an experimental task in kinematic studies [28]. During each trial, the participant was asked to provide a VAS score while lifting the object. We recorded the participant’s trunk movement using a three-dimensional (3D) motion capture system (KinemaTracer; KisseiComtec, Matsumoto, Japan).

### Outcome measurements

The following questionnaires were used to determine the study outcome.

#### VAS

As noted above, the participants were asked to rate their maximum pain in the past 4 weeks and their pain during the movement task on the VAS. For the VAS scoring, the participant was presented with a sheet of paper with a 100-mm line, and it was explained that 0 mm (the left end) represented no pain, while 100 mm (the right end) indicated severe pain. The participant was asked to mark the sheet according to the level of pain they felt. They rated their pain over the past 4 weeks while resting in a seated position before performing the experimental task, and then again immediately after completing the experimental task.

#### TSK

Fear of movement was assessed by the TSK [22], a 17-item questionnaire that assesses fear of movement, with higher scores reflecting greater fear. The participants rated how much they agreed with each of the 17 statements, and the ratings were summed to yield a total score ranging from 17–68, with a cut-off value of 37 points [29].

#### PCS

The participant’s catastrophic thinking was assessed using the PCS, a 13-item self-reported questionnaire that evaluates items in three dimensions: rumination, helplessness, and magnification [23]. The PCS scores reveal a respondent’s thoughts and perceptions regarding pain in a variety of experiences, with higher scores indicating greater levels of catastrophizing. Here, the cut-off value is 30 points [30].

#### PASS-20

Anxiety related to pain was assessed with the PASS-20, a 20-item measure of pain-related anxiety [24]. For each item, the respondent indicates the degree to which they agree with statements regarding how they respond to or think about pain. Responses are made on a 5-point scale.

#### RDQ

We used the RDQ to assess disability directly related to LBP. The RDQ is a 24-item questionnaire with a dichotomous scoring format: yes (= item is applicable), or no (= item is not applicable). Scores can vary from 0 (no disability) to 24 (severe disability) [25]. The RDQ is a reliable and valid instrument for measuring disability related to LBP [31].

#### LBP severity

To evaluate the severity of LBP, Von Korff’s grading was used as follows: grade 0 was defined as no LBP; grade 1 as LBP that does not interfere with work; grade 2 as LBP that interferes with work but does not cause absences; and grade 3 as LBP that interferes with work, leading to sick-leave [26].

#### Experimental task

As the experimental task, the participants were asked to lift an object five times with a 1-min interval after each lifting (Trials 1–5). The object consisted of a 520×365×305-mm box placed on the ground and weighing 37.5% of participant’s body weight, as it an earlier study [32]. That study indicated that the positional relationship between the subject’s feet and the box affected the low back load during lifting [33]. Hence, in the present experiment, the participant’s start position was standing with the feet shoulder- width apart, and the centerline of the box width was placed to match the center point between the feet. The box was positioned at one half the length of the foot from the toe. The participants were asked to perform the lifting task at a comfortable speed, and to lift the box to waist-height. A 1-min rest was applied between trials. During the rest period after each trial, the participants indicated their pain intensity on the VAS.

#### Kinematic data collection and processing

**Fig 1:**
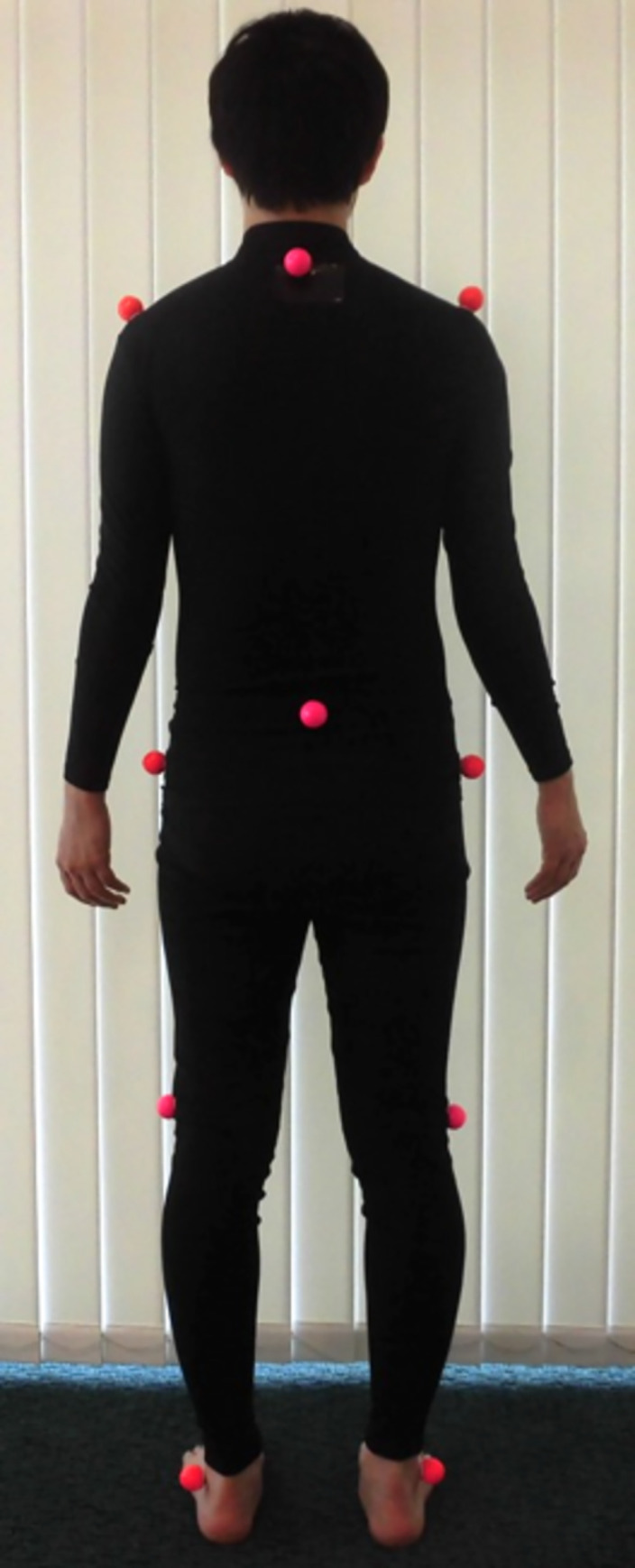
The configuration of motion markers. Ten 30-mm-dia. markers were attached to the participant’s thoracic spine (Th1 spinous process) and pelvis (S2 spinous process), and bilaterally on the acromion, great trochanter, lateral femoral epicondyle, and lateral malleolus.

**Fig 2:**
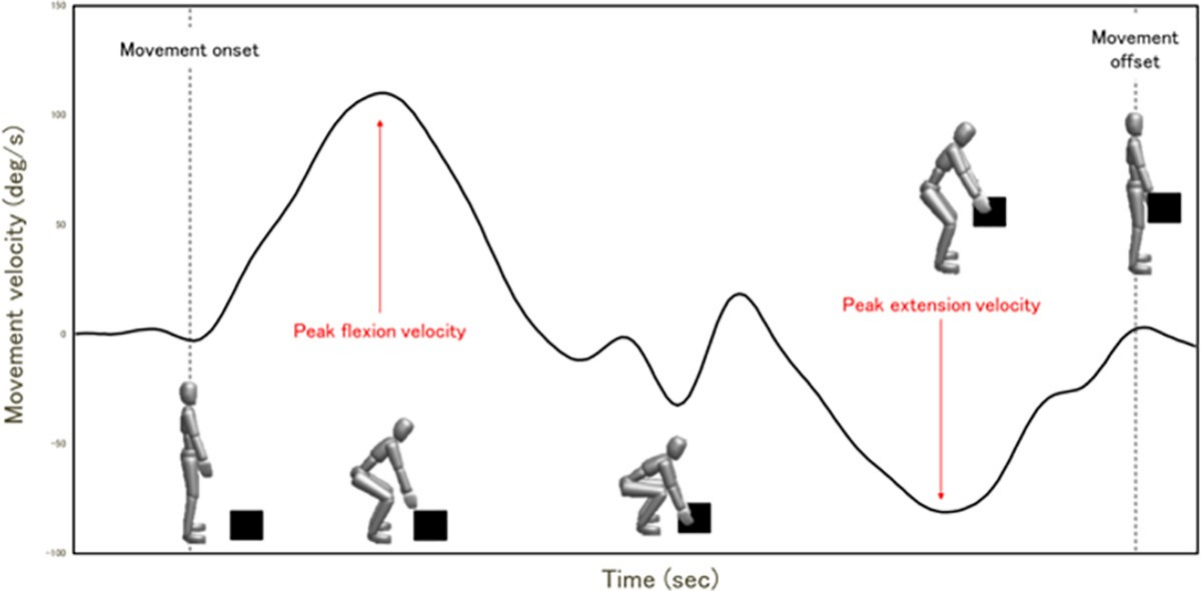
Time series variation of the velocity of trunk movement during lifting. The kinematic data were extracted from the peak trunk flexion velocity and the peak trunk extension velocity.

A 3D motion capture system including a four-charge-coupled device (CCD) camera was used to record 3D marker displacement at a sampling frequency of 60 Hz: a total of 10 30-mm-dia. markers were attached to the participant’s thoracic spine (Th1 spinous process) and pelvis (S2 spinous process) and bilaterally on the acromion, great trochanter, lateral femoral epicondyle, and lateral malleolus (Fig 1). The recorded kinematic data were low-pass filtered with a second-order recursive Butterworth filter with a cutoff frequency of 6 Hz, following the technique reported by Katsuhira [33]. In addition, to define the trunk angle, a vector was created using markers placed on the Th1 spinous process and S2 spinous process. The trunk angle was calculated using the angle between the vector joining these markers and the vertical axis. The motions used to perform the experimental task were primarily in the sagittal plane; we thus used the peak trunk flexion velocity and peak trunk extension velocity (Fig 2).

### Statistical analysis

After using the Shapiro-Wilk test to confirm the normality of the participants’ age, height, body weight, and kinematic parameter data, we compared the age, height, body weight, and sex distribution between the LBP and control groups using the Mann-Whitney U-test and Fisher’s exact test.

To compare differences in kinematic features between the LBP and HC groups, we used a generalized linear mixed model (GLMM), which provides a more sophisticated analysis method than a traditional repeated measures analysis of variance (ANOVA) because it accounts for random effects such as differences among individuals, which is not possible with a traditional ANOVA. The response variable was the kinematic parameters, and the fixed effects were the trial condition (Trials 1–5), group factors (LBP group vs. HC group) and their interaction. For the random effect, we selected the participant ID for differences among individuals.

In addition, to test our hypothesis, we performed a mediation analysis to analyze the relationships among pain, the kinematic parameters, and fear of movement. We created a mediation model with X (independent variable: kinematic parameter)-M and (mediator variable: TSK)-Y (dependent variable: VAS). Per the model described by Baron and Kenny [34], the following a priori steps had to be successfully met: (a) Variable X is significantly associated with variable Y. (b) Variable X is significantly associated with variable M. (c) Variable M is significantly associated with variable Y. Spearman’s rank correlation coefficients were obtained as a prelude to the mediation analysis, and we confirmed whether each variable met these assumptions.

A bootstrap sampling procedure was used to determine the significance of indirect effects. In the present study, we specified 1,000 bootstrap iterations and estimated the 95% confidence intervals (CIs). The statistical analyses were performed using R ver. 2.8.1 and HAD ver. 14.8 [35], and the level of significance was set at 5%.

## Results

### Participant characteristics and clinical information

Table 1 summarizes the participants’ characteristics. There were no significant differences between the LBP and HC groups in age, height, or body weight. In the evaluation of pain-related indicators, only the TSK scores (39.0±3.8 points) exceeded the cut-off point (37.0 points) in the LBP group. Von Korff’s grades for LBP severity were grade 1 (85.7%) and grade 2 (14.3%); none of the participants required an absence from work.

**Table 1:** Participants’ characteristics and clinical information.

	HC group	LBP group
	(n = 20)	(n = 35)
Age, yrs	28.0 (24.0–30.0)	30.0 (25.0–32.0)
Males/Females, n (%)	18 (90)/2 (10)	26 (74.3)/9 (25.7)
Height, cm	171.0 (168.0–174.3)	170.0 (164.0–174.0)
Weight, kg	64.5 (59.5–71.0)	62.0 (56.5–67.5)
Duration, months *	0 (0)	60.0 (18.0–60.0)
Pain intensity in the past 4 weeks (VAS: 0–100) *	0 (0)	28.0 (13.5–51.5)
Pain intensity during the experimental task (VAS: 0–100)	0 (0)	0 (0)
TSK (17–68) *	24.5 (22.0–27.0)	39.0 (36.0–40.5)
PCS (0–52) *	5.0 (4.0–7.5)	18.0 (16.0–22.0)
PASS-20 (0–100) *	19.0 (17.0–21.0)	34.0 (24.5–43.5)
RDQ (0–24) *	0 (0)	2.0 (1–3)
Severity of LBP, n (%) *	Grade0: 20 (100)	Grade1: 30 (85.7)
		Grade2: 5 (14.3)

Values are median (interquartile range) or n (%).

*p<0.01 between LBP and HC. PASS-20: Pain Anxiety Symptoms Scale-20, PCS: Pain Catastrophizing Scale, TSK: Tampa Scale for Kinesiophobia, VAS: Visual Analogue Scale.

### Comparison of kinematic parameters between the groups

Table 2 provides the average data (standard deviation [SD]) of the participants’ peak trunk flexion and peak trunk extension velocity while lifting the object, and Table 3 lists the GLMM results for their kinematic data. The peak trunk extension velocity showed a significant main effect for the group factors [95%CI: 6.94, 29.08] and their interactions [95%CI: −22.27, −1.21].

**Table 2:** The kinematic data for peak trunk flexion and peak trunk extension velocity.

		Trial 1	Trial 2	Trial 3	Trial 4	Trial 5
Flexion velocity, deg/s	HC	95.30 (26.60)	103.52 (29.78)	104.08 (27.47)	111.25 (22.16)	111.51 (23.95)
	LBP	97.25 (25.26)	103.27 (21.01)	106.98 (22.29)	110.41 (24.13)	112.16 (19.49)
Extension velocity, deg/s	HC	−88.69 (12.53)	−91.27 (20.45)	−92.95 (20.48)	−94.23 (21.19)	−97.20 (20.45)
	LBP	−70.93 (19.60)	−85.01 (21.82)	−88.46 (22.22)	−90.36 (23.07)	−89.18 (22.27)

Values are mean (SD).

**Table 3:** Results of comparison kinematic features between the groups.

	Estimate	Est. Error	l-95%CI	u-95%CI	Rhat
Flexion velocity:					
Intercept	95.72	5.90	83.90	107.29	1.00
Group	1.57	7.55	−12.80	16.30	1.00
Trial	8.27	3.23	1.95	14.88	1.00
Group-Trial	−2.29	4.01	−10.52	5.36	1.00
Extension velocity:					
Intercept	−88.73	4.52	−97.65	−79.80	1.00
Group	17.83	5.68	6.94	29.08	1.00
Trial	−2.51	4.27	−10.88	5.77	1.00
Group-Trial	−11.57	5.40	−22.27	−1.21	1.00

**Fig 3:**
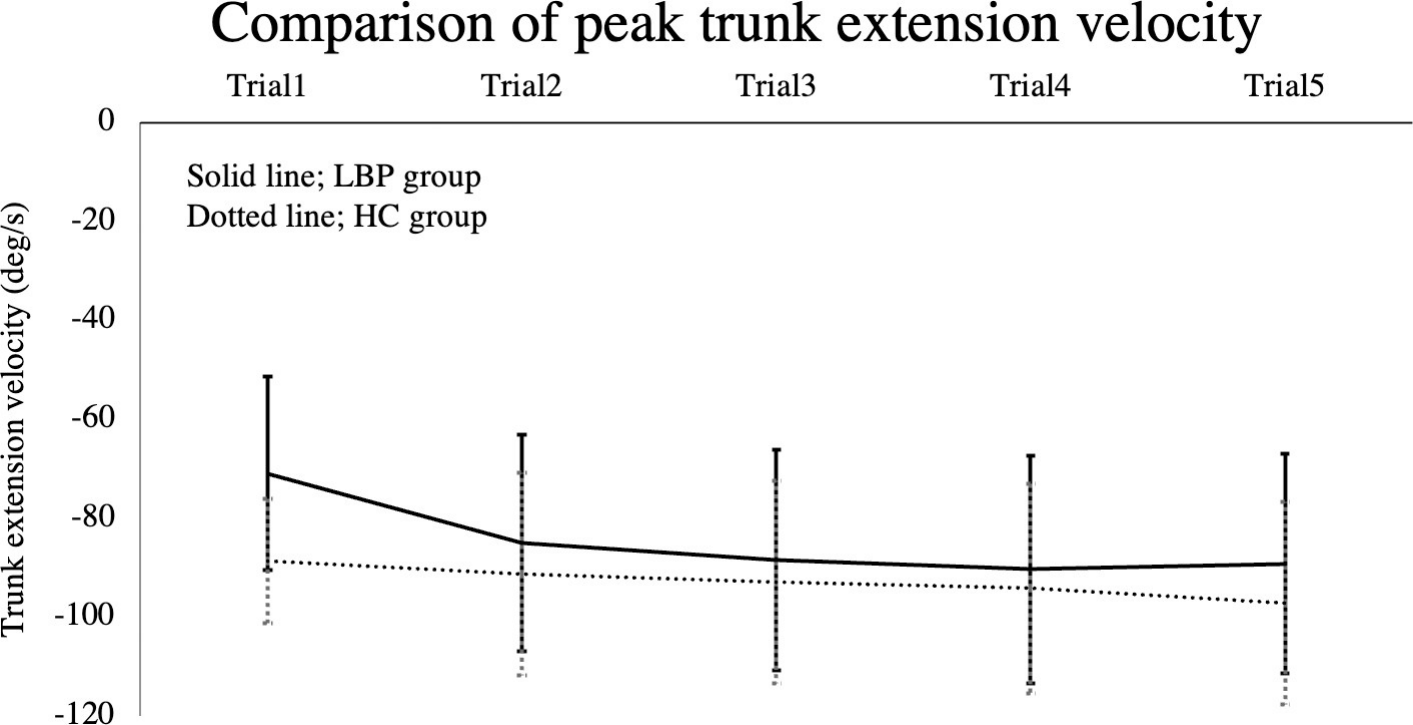
Comparison of peak trunk extension velocity at each trial in the two groups. The LBP group showed slower trunk extension in Trial 1 compared to the HC group.

**Fig 4:**
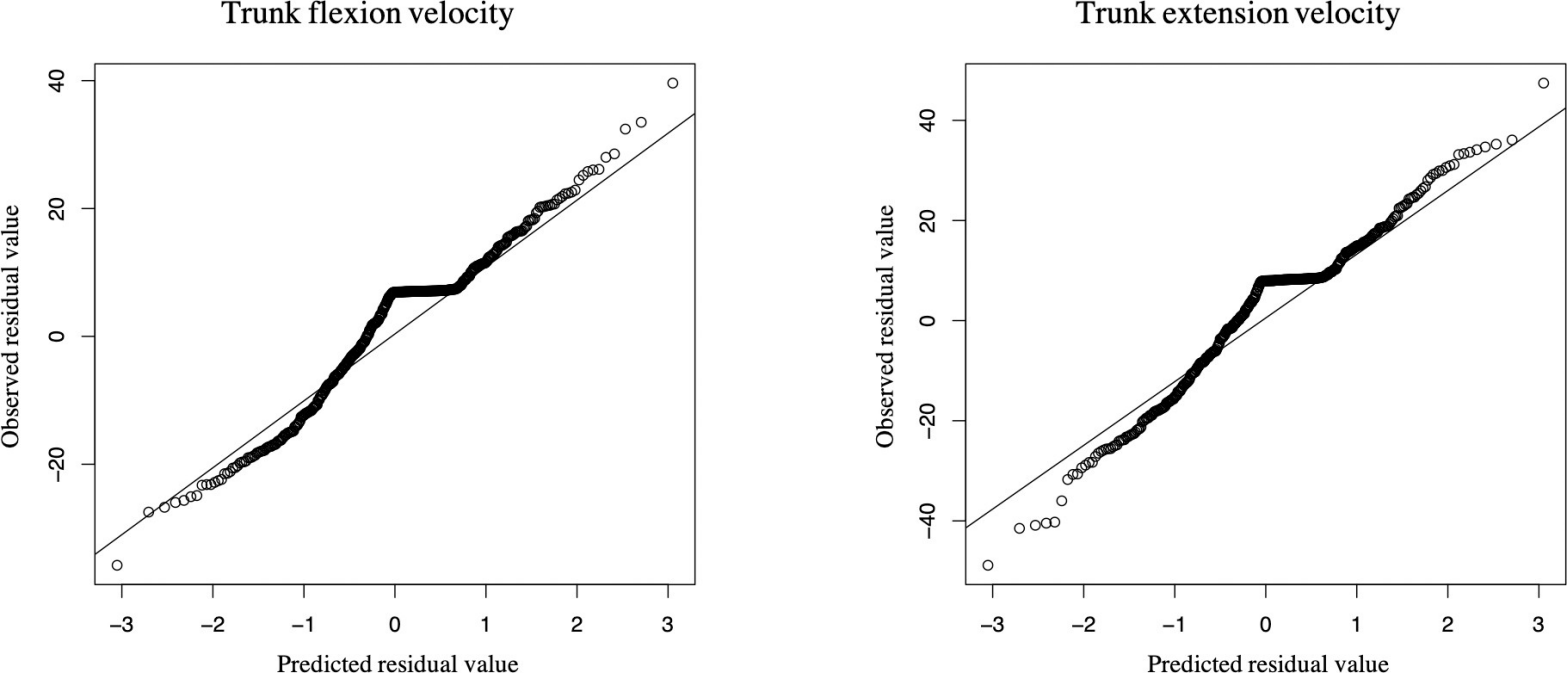
Residual plots of the GLMM. The Plot of each parameter approaches a 45° angle with residuals normally distributed, indicating that the quality of the GLMM is ensured.

Fig 3 illustrated the changes the participants’ peak trunk extension velocity at each trial in both groups, and the results indicate that trunk extension was particularly slow in Trial 1 in the LBP group compared to the HC group. In contrast, the peak trunk flexion velocity showed no significant main effect for the group factor [95%CI: −12.80, 16.30] or interactions [95%CI: −10.52, 5.36], although there was a significant main effect for the trial condition [95%CI: 1.95, 14.88]. Fig 4 provides the residual plots of each GLMM. The plot of each parameter approaches a 45° angle with residuals normally distributed, indicating that the quality of GLMM is ensured.

None of the participants in the LBP group experienced pain during the experimental task.

### Mediation analysis

We first determined the results of correlation analyses as a prelude to the mediation analysis; the results are presented in Table 4. In these analyses, we focused on the peak trunk extension velocity in the first trial, where there was a notable difference between the LBP and HC groups. The peak trunk extension velocity was significantly correlated with pain intensity over the past 4 weeks and with the TSK scores, but not with the PCS, or PASS-20 (Table 4). The participants’ pain intensity during the 4 weeks prior to the study was significantly correlated with the TSK scores (r = 0.41, p<0.05) and PCS scores (r = 0.36, p<0.05).

**Table 4:** The relationships between the kinematic data at the first trial and pain-related factors.

	VAS	TSK	PCS	PASS−20
Flexion velocity, deg/s	−0.12	−0.10	−0.30	−0.02
Extension velocity, deg/s	0.34*	0.62**	0.18	0.23

The data are r-values. VAS: pain intensity in the past 4 weeks.

*p<0.05

** p<0.01.

**Fig 5:**
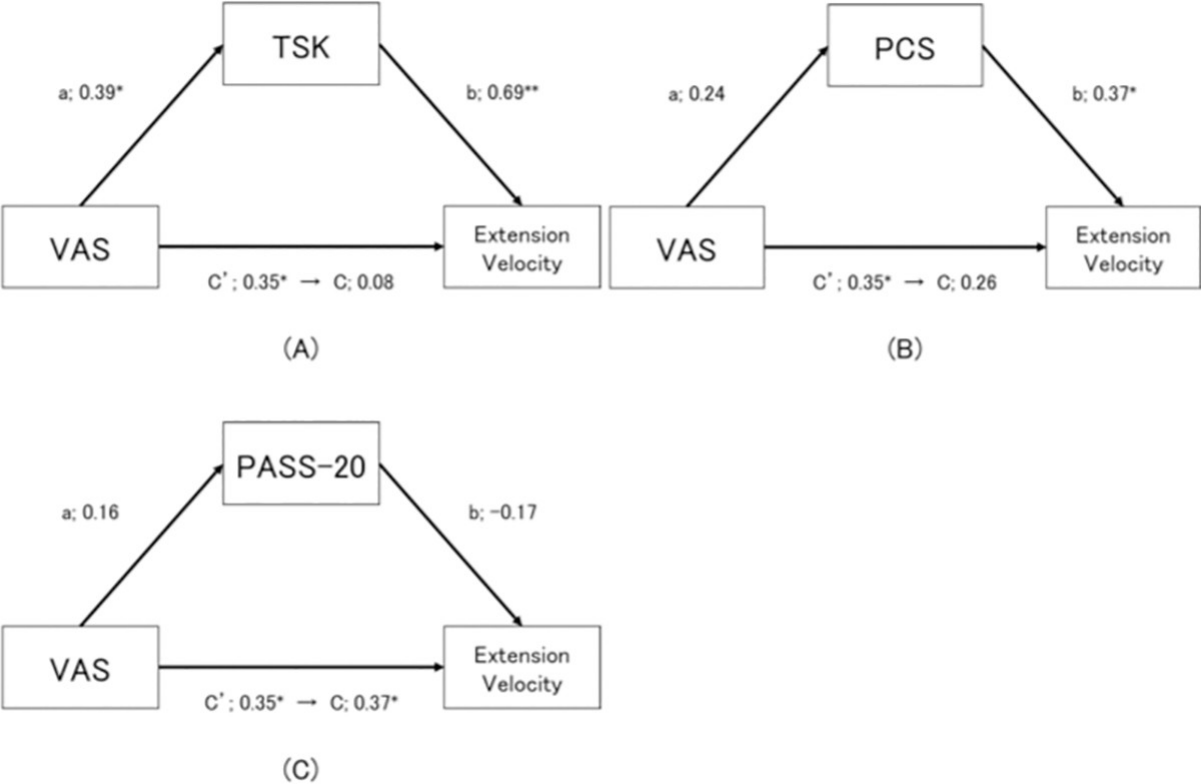
Psychological factors that mediated the relationship between the participants’ VAS scores for pain in the past 4 weeks and the peak trunk extension velocity. These images were created using the data from Trial 1. A: The TSK is the mediated variable. B: The PCS is the mediated variable. C: The PASS-20 is the mediated variable. *p<0.05, **p<0.01.

Next, based on the results of the correlation analyses, we performed a mediation analysis to investigate whether fear of movement mediated the relationship between pain intensity in the past 4 weeks and the kinematic parameters. The tested model is illustrated in Table 5. The results of the TSK model revealed a significant positive association between pain intensity in the past 4 weeks and the peak trunk extension velocity, without a moderator. However, with the introduction of the TSK, there was no significant association between pain intensity in the past 4 weeks and peak trunk extension velocity, although we observed significant positive associations between pain intensity in the past 4 weeks and the TSK, and between the TSK and peak trunk extension velocity (Fig 5).

**Table 5:** The results of the mediation analysis.

Path/effect		β	SE	p-value/95% BCCI
a	Pain VAS ⇒ TSK	0.39	0.03	0.02
b	TSK ⇒ Peak velocity	0.69	0.58	0.01
c	(direct effect) Pain VAS ⇒ Extension velocity	0.08	0.12	0.55
c’	(total effect) Pain VAS ⇒ Extension velocity	0.35	0.15	0.04
a×b	(indirect effect) Pain VAS ⇒ Extension velocity	0.27	0.13	(LL = 0.07, UL = 0.56)
a	Pain VAS ⇒ PCS	0.24	0.10	0.17
b	PCS ⇒ Peak velocity	0.37	0.26	0.02
c	(direct effect) Pain VAS ⇒ Extension velocity	0.26	0.15	0.11
c’	(total effect) Pain VAS ⇒ Extension velocity	0.35	0.15	0.04
a×b	(indirect effect) Pain VAS ⇒ Extension velocity	0.09	0.07	(LL = −0.01, UL = 0.30)
a	Pain VAS ⇒ PASS-20	0.16	0.10	0.36
b	PASS-20 ⇒ Extension velocity	−0.17	0.26	0.32
c	(direct effect) Pain VAS ⇒ Extension velocity	0.37	0.16	0.03
c’	(total effect) Pain VAS ⇒ Extension velocity	0.35	0.15	0.04
a×b	(indirect effect) Pain VAS ⇒ Extension velocity	−0.03	0.05	(LL = − 0.21, UL = 0.02)

BC: bias corrected, LL: lower limit, SE: standard error, UL: upper limit.

There was also an indirect effect of pain intensity in the past 4 weeks and the peak trunk extension velocity via TSK, as we obtained a bootstrap CI (95%CI) of 0.07–0.56 and to exhibit a significant positive effect. Other pain-related factors showed no significant effect.

## Discussion

We conducted this study to identify impaired trunk movement during work-related activity in individuals with LBP and to investigate whether the abnormalities were caused by a generalization of fear of movement-related pain. The results of our object-lifting experiment demonstrated that (1) the participants with LBP had reduced trunk extension movement velocity while lifting an object, and (2) impaired trunk movement was not related to sensory pain but was related to fear of movement based on past pain experiences. In other words, our results provide evidence of an association between generalized fear of movement-related pain and the impaired trunk movement in individuals with LBP.

We first determined the kinematic differences during lifting between the LBP and HC groups. The participants in the LBP group had reduced velocity of trunk extension movement in Trial 1, which is inconsistent with a study that found no difference in lumbar velocity in subjects with chronic lower back pain [18]. A possible reason for this inconsistency may be the difference in instructions given to the participants during the experimental task. In the previous study [18], the participants were instructed to move their trunk as quickly as possible, whereas we instructed the present study’s participants to move their trunk at a comfortable pace. These different instructions would presumably have influenced the trunk velocity during the object-lifting trials. Slower trunk movement is thought to minimize the pain caused by mechanical stress in the lumbar structure, and movement error is a form of excessive protective behavior in individuals with LBP [18, 19]. Our present observation of a decrease in the velocity of trunk extension movement might therefore be interpreted as a compensatory movement to reduce the mechanical load on the lumbar spine, because the mechanical load reaches a maximum during the trunk extension phase [33].

Psychological factors such as fear of movement may affect the impaired trunk movement in individuals with LBP [18, 19]. These studies reported that impaired trunk movement was associated with fear of movement; however, only a simple correlation analysis of the association between impaired trunk movement and fear of movement was conducted in the studies [18, 19]. The precise relationship between fear of movement and impaired trunk movement thus remains to be established. In our present study’s LBP group, although some participants had experienced pain in the prior 4 weeks, none complained of pain during the experimental task.

The results of the mediation analysis also revealed that the relationship between pain intensity in the prior 4 weeks and the peak angular velocity of trunk extension in Trial 1 was completely mediated by the TSK. This suggests that the characteristics of impaired trunk movement in individuals with LBP is not due to the pain that occurs during movement, but rather to the fear of movement that is caused by past pain experiences. The existing research indicates that pain can be considered a threat signal, which itself triggers protective responses such as psychophysiological arousal (e.g., the startle response and increased muscle tone) and escape behavior. For example, if pain occurs repeatedly during a particular movement, that movement itself may come to signal bodily harm, elicit fear of movement, and spur avoidance behavior [16]. Meulders argued that fear of movement not only occurs when the individual performs painful movements but also spreads through contextually relevant movements related to a past pain experience, which they described as the generalization of fear of movement-related pain [16]. The present data support this concept. Our results suggest that pain that occurred in the past during work-related activity caused a generalized fear of movement-related pain, such as a fear that "pain may occur by trunk extension", and thereby decreased the LBP participants’ velocity of trunk extension during the lifting of the test object. In other words, the slower trunk extension movement may be caused by the generalization of fear of movement-related pain based on past pain experience.

Our participants also exhibited an increase in movement speed after the second trial. This result differs from that of a study in which the lumbar velocity in subjects with LBP was significantly impaired, regardless of the number of trials [19]. One possible reason for these discrepant results may be differences in the severity of LBP between our participants and those of the previous study. In that investigation [19], the subjects were patients with severe LBP being treated at medical facilities. The present participants in the LBP group had mild symptoms and reported that their work was manageable. This suggests that our participants with mild LBP may have adapted to their condition in stages, slowing the velocity of their trunk extension movement due to fear of movement-related pain from a novel activity in the first trial, and then, in the second trial, increasing their velocity of trunk extension movement because no pain occurred in the first trial.

In an actual work situation, it is assumed that generalization of fear of movement-related pain is likely to affect impaired trunk movement, because workers must handle heavy objects of various weights. In such a case, it is possible that fear of movement is triggered and leads to excessive avoidance behaviors (e.g., reduced trunk extension movement velocity), despite the individual being in a safe situation without pain. Fear of movement is a particularly robust predictor of LBP disability [36], such as abnormal trunk movement during work-related activity, and it could be one of the causes of prolonged LBP.

Despite the relationship between slower trunk extension movement and fear of movement, we observed that the trunk mechanical properties were not associated with pain catastrophizing (i.e., the PCS scores) or anxiety (the PASS-20 scores). This finding might be attributable to properties of the questionnaires themselves. The TSK contains items pertaining to physical activity and exercise (e.g., pain could increase or re-injury could occur if the respondent increases his/her physical activity or exercise level). In contrast, the PCS and PASS-20 primarily contain questions related to beliefs about pain and painful physical symptoms. An examination of the relationship between impaired movement and psychological factors identified a higher correlation for the TSK compared to the PCS and PASS-20 [37]. The TSK might therefore be more sensitive to the physical demands of lifting.

The results of our kinematic study quantified the influence of the generalization of fear of movement-related pain in workers with LBP. Particularly with respect to trunk extension movement during the lifting of an object, if the movement is slow, clinicians should consider the influence of generalization of fear of movement-related pain. Our findings also suggest that intervention for generalization of fear of movement-related pain is needed to improve impaired trunk movement in individuals with LBP.

### Study limitations

This study has several limitations. First, the outcomes of fear of movement were merely those measured by the TSK; task-specific fear was not assessed. Second, the sample size was relatively small (55 participants). The GLMM and the mediation analysis (bootstrap sampling procedure) enables stable estimations even with a small sample size [38–40], but further research with more subjects might be necessary to arrive at a definite conclusion. Third, the sample size of the HC group (n = 20) was smaller than that of the LBP group (n = 35), because the inclusion criteria for the HC group were strict. The difference in sample size between the two groups might have affected the results. Fourth, because kinetic and electromyographic variables were not used in this study, the lumbar load during the lifting task in not known. Finally, there was no significant difference between the LBP and HC groups in the sex distribution, but there was a slightly higher percentage of women in the LBP group. The difference in attributes between the LBP and HC groups might thus have affected the results.

### Future directions

Considering that this was a cross-sectional study, it is a preliminary investigation. Future research should address the limitations of this study (e.g., the sample size and sample bias) and be based on a more detailed study design. e.g., regarding the assessment of fear of movement and the movement analysis method.

Trunk flexion and extension velocity are aspects of kinematics, and the trunk mechanical properties were unknown in the present study design. The mechanical load on the trunk that occurs during work-related activity has a direct effect on LBP symptoms. In future work, the incorporating of kinetic and electromyographic variables may clarify whether slower trunk extension movement is a harmful movement error against LBP symptoms or a rational compensatory movement.

In addition, although the outcomes of fear of movement were measured by the TSK only in this study, the assessment of task-specific fear is also recommended in future investigation. An earlier study revealed the existence of individuals with LBP who feel threatened only by particular movements, although their TSK scores were low [41]. We need to incorporate not only the TSK but also the measurement of task-specific fear by using pictures of work-related activity as an outcome of fear of movement in a future study [42].

Finally, our present findings suggest the need for interventions that reduce fear of movement, which is generalized by past pain experiences, in order to improve impaired trunk movement in individuals with LBP. In other words, the results of this study could be noteworthy as a target for future research, including the designs of intervention methods regarding interaction between generalized fear and trunk movement. It has been reported that LBP involves a combination of psychological factors and impaired trunk movement that becomes severe [43]. Therefore, comprehensive interventions that include both trunk movement and psychological factors may lead to much greater improvements of LBP symptoms and work-related disability. It is necessary to examine, based on longitudinal data, whether the combination of psychosocial interventions (such as pain neurophysiology education) and exercise (such as stretching) would improve the impaired trunk movement as well as reduce the generalization of fear of movement-related pain.

## Conclusion

We sought to identify impaired trunk movement during work-related activity in individuals with LBP, and to investigate whether the abnormalities were caused by a generalization of fear of movement-related pain. The results of a mediation analysis demonstrated that the relationship between pain intensity in the prior 4 weeks and the peak angular velocity of trunk extension was completely mediated by the TSK in the first object-lifting trial. The impaired trunk movement during work-related activity in our participants with LBP was therefore not related to sensory pain during the movement tasks but rather was related to the generalization of fear of movement-related pain. Impaired trunk movement was observed even in the present participants with mild, manageable low back pain, and it might thus be necessary to reduce fear of movement rather than sensory pain to improve the movement disorders of the trunk.
